# A cryptic balanced translocation (5;17), a puzzle revealed through a critical evaluation of the pedigree and a FISH focused on candidate loci suggested by the phenotype

**DOI:** 10.1186/s13039-015-0172-1

**Published:** 2015-09-02

**Authors:** A. Primerano, E. Colao, C. Villella, M. D. Nocera, A. Ciambrone, E. Luciano, L. D’Antona, M. F. M. Vismara, S. Loddo, A. Novelli, N. Perrotti, Paola Malatesta

**Affiliations:** Unit of Medical Gentics, University Hospital Mater Domini, Catanzaro, Italy; Mendel Laboratory, Casa Sollievo della Sofferenza Hospital, IRCCS, San Giovanni Rotondo, Italy; Genetics Unit, Bambino Gesu Children’s Hospital, IRCCS, Rome, Italy; Department of “Scienze della Salute”, Università UMG di Catanzaro, Catanzaro, Italy

## Abstract

We report a case of a woman with a cryptic balanced translocation between chromosomes 5 and 17, suspected during genetic counseling.

The woman had a history of previous fetal losses attributed to lissencephaly and intra uterine growth retardation (IUGR) and a daughter with dysmorphic features and mental retardation, previously attributed to a small deletion 5pter, detected years ago by a first generation CGH-array. This peculiar combination of personal and family history suggested the opportunity to carry out a FISH approach, focusing on chromosomes 5 and 17, based on the idea that a malsegregation secondary to a balanced translocation, might have escaped the first CGH array. This approach allowed the identification of a balanced translocation in the mother, FISH in the affected child confirmed the partial 5p deletion predicted by the previous CGH array and identified a new 17p duplication that had not been detected before. The described translocation opens the possibility of alternative imbalances that were probably responsible for previous fetal losses. The imbalances were confirmed by a new high resolution SNP array.

We conclude that despite the availability of highly effective and sensitive genomic approaches a careful evaluation of medical history is highly recommended since it can suggest clinical hypothesis that can be confirmed by more classical and molecular cytogenetic based approaches.

## Background

Approximately 5 % of the general population is estimated to be carrier of a balanced rearrangement. Derivative chromosomes are probably more common than expected since they may appear normal at standard resolution karyotype. Although balanced translocation are associated with infertility and recurrent miscarriages, the clinical consequences of unbalancing of derivative chromosomes can be extremely serious ranging from severe phenotypes to lethality. The limit for the detection of genomic rearrangements by karyotype is estimated to be 5–10 Mb at the 500– 550 band level, at least in regions where the band pattern is distinctive [1].

More recently the use of microarrays provided a powerful tool for studying the entire genome for copy number variations by comparative genomic hybridization (CGH-array) and for the distribution of single nucleotide polymorphisms (SNP-array) [2]. The birth of a child with a derivative chromosome and unbalanced karyotype represents a painful and difficult event for the family, but may also offer the opportunity to link genotype and phenotype and to infer the functional consequences of genomic imbalances.

In the present report we describe how a careful evaluation of the medical history of the proband and her family allowed the identification of an unusual balanced translocation, that was responsible for different phenotypes in one daughter and in two fetuses.

## Case presentation

The couple came to our attention for genetic counseling to define their reproductive risk. A standard karyotype performed previously was reported normal in both the members of the couple. The woman and her male partner had four pregnancies including two abortions.

The first occurred at the 34^th^ week of gestation for complete lissencephaly and corpus callosum agenesis (assessed by fetal MRI); karyotype of abortion material was reported normal (46,XX). The second abortion occurred at 9^th^ week-gestation for IUGR. One pregnancy resulted in a phenotypically normal son and another one, conceived when the mother was 33-years old and the father 34, resulted in an affected baby girl.

She was born at term by caesarian section because of mother’s pelvis altered conformation. At birth, physical examination revealed laryngeal stridor, feeding problems due to difficulties in swallowing and sucking, together with mild dismorphic traits. Neonatal screening for inborn errors of metabolism revealed congenital hypothyroidism. In the first six months, a delayed motor development was recorded. Speech was also delayed until the age of 30 months, and sphincterial control was reached at 4 years. Based on the laryngeal stridor, mild dysmorphism, and developmental delay, the baby was studied, between 6 and 18 months, by standard karyotype, FISH for 5p15.2 to rule out Cri du chat syndrome and CGH array [Agilent 105A, 20 Kb resolution, CGH Analytics software (v3.4.40)]. The analysis were not diagnostic for Cri du chat but revealed, instead a 4.58 MB deletion in 5p15.33, a little distal to the classical cri du chat region.

The child came to our attention for the first time when she was six years old. She had pink skin and some loose, fluffy hair on the back. The mouth was small and a wandering rash was visible on the tongue. Teeth appeared overcrowded, although teething was reported normal. Upon physical examination a holosystolic murmur, like a whiff, was heard best at the apex, extremities, chest and abdomen were normal .

**Fig. 1:**
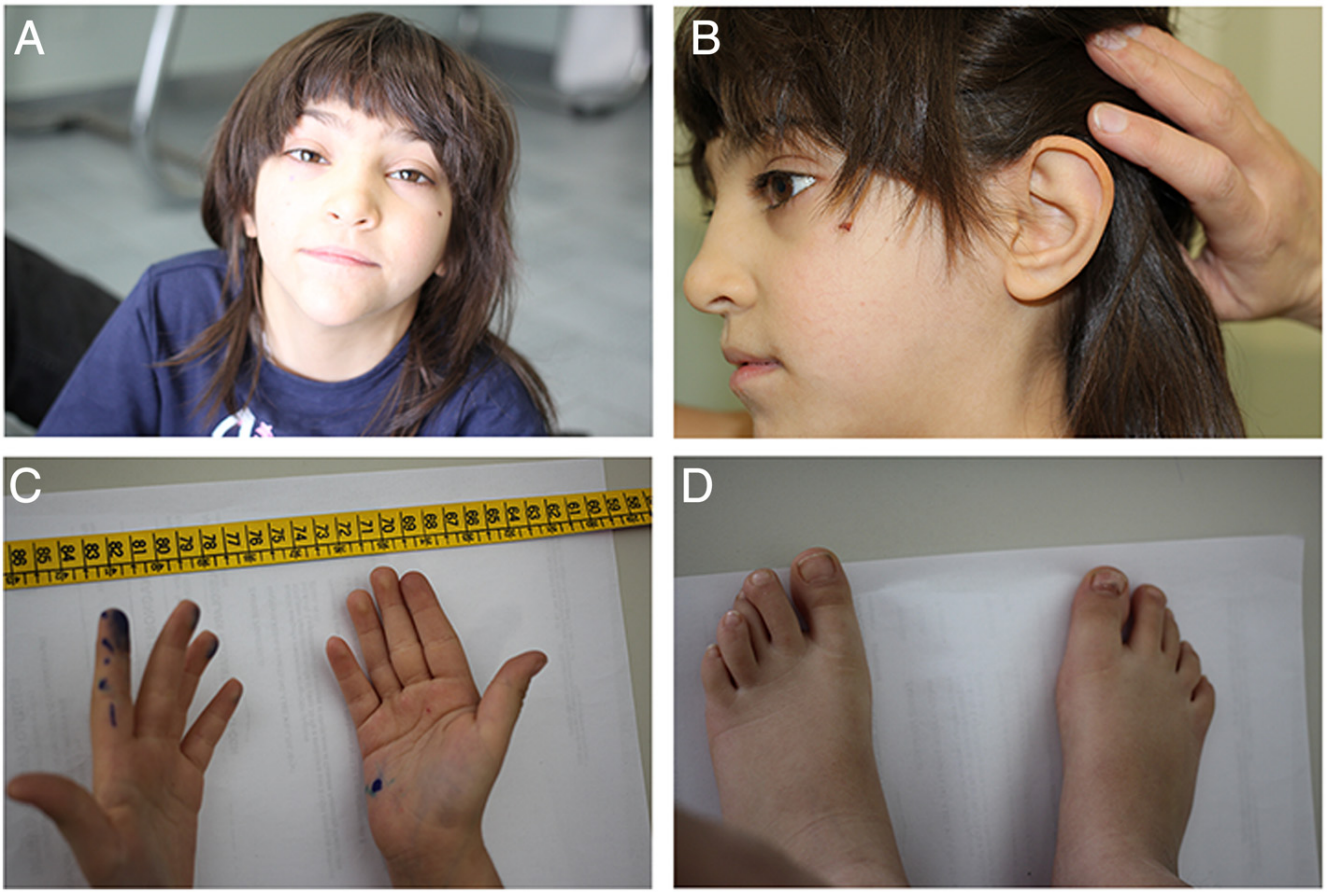
**a** The child had long triangular shaped face, narrow forehead with a lower front hairline, mild synophrys, broad nasal bridge, a small mouth and a horizontal labial fissure. **b** Low set prominent ears posteriorly rotated with abnormally folded anthelices, (**c**) Abnormal dermatoglyphics, with long three finger crease (TFC), incomplete five finger crease (FFC) (starts under the fouth finger and end on radial side), short thumb crease (TC). short palm. **d** Short toes with clinodactily of the fourth and second left toes

The child had long triangular shaped face, narrow forehead with a lower front hairline, mild synophrys, low set prominent ears posteriorly rotated with abnormally folded anthelices, broad nasal bridge, horizontal labial fissure, abnormal dermatoglyphics and clynodactly with overlapping toes. Some of the clinical features can be recognized in Fig. 1.

## Methods

### Standard karyotype

Classical high resolution GTG chromosome banding was performed according to standard procedure. Briefly peripheral blood was collected in heparin vacutainers (Becton Dickinson, Franklin Lakes, NJ, USA). Whole blood (0.5 mL) cultures were set up in10 mL RPMI 1640 media (Technogenetics srl, Milano, Italia), containing 20 % fetal calf serum (Euroclone spa, Milano, Italia), antibiotic mixture 10,000 IU/ml (Euroclone spa, Milano, Italia) and phytohemagglutinin M (PHA) (Technogenetics srl, Milano, Italia). Chromosomes from (PHA)-stimulated peripheral blood lymphocytes were analyzed by Giemsa-Trypsin-Giemsa (GTG)-banding. karyotypes were interpreted using the recommendation of the International System for Human Cytogenetic Nomenclature (2013).

**FISH** on metaphase plates was performed according to previously published methods [3], using the following probes: Miller-Dieker probe (Abbott) and subtelomeric probe for 5p (kit ToTelVysion from Vysis) according to the manufacturer’s instruction.

### SNP-array

Genomic DNA was extracted from blood samples using the DNA blood extraction kit (Nuclear Laser Medicine srl, Milano, Italia) according to the manufacturer’s instructions. DNA concentration was measured with a NanoDrop™ Spectrophotometer. Microarray analysis was performed using CytoScan HD (Affymetrix, Inc, Santa Clara, CA, USA), according to the manufacturer’s instructions, and the data were analyzed by Chromosomes Analysis Suite Software(Chas) v2.1 (release hg19) (Affymetrix, Inc, Santa Clara, CA, USA).

## Results

The reproductive history of the couple as well as the phenotype of the affected baby girls were peculiar. The mother had experienced two abortions, one for complete lissencephaly and the other for IUGR. The affected baby girl had a complex phenotype that had been attributed to a 4.58 MB deletion in 5p15.33. We hypothesized that the occurrence of lissencephaly and segmental deletion of 5p15.33 in the same kindred could be attributed to the presence of an undiscovered parental balanced translocation that resulted in unbalancing of either chromosome 5 or 17 during meiosis.

**Fig. 2:**
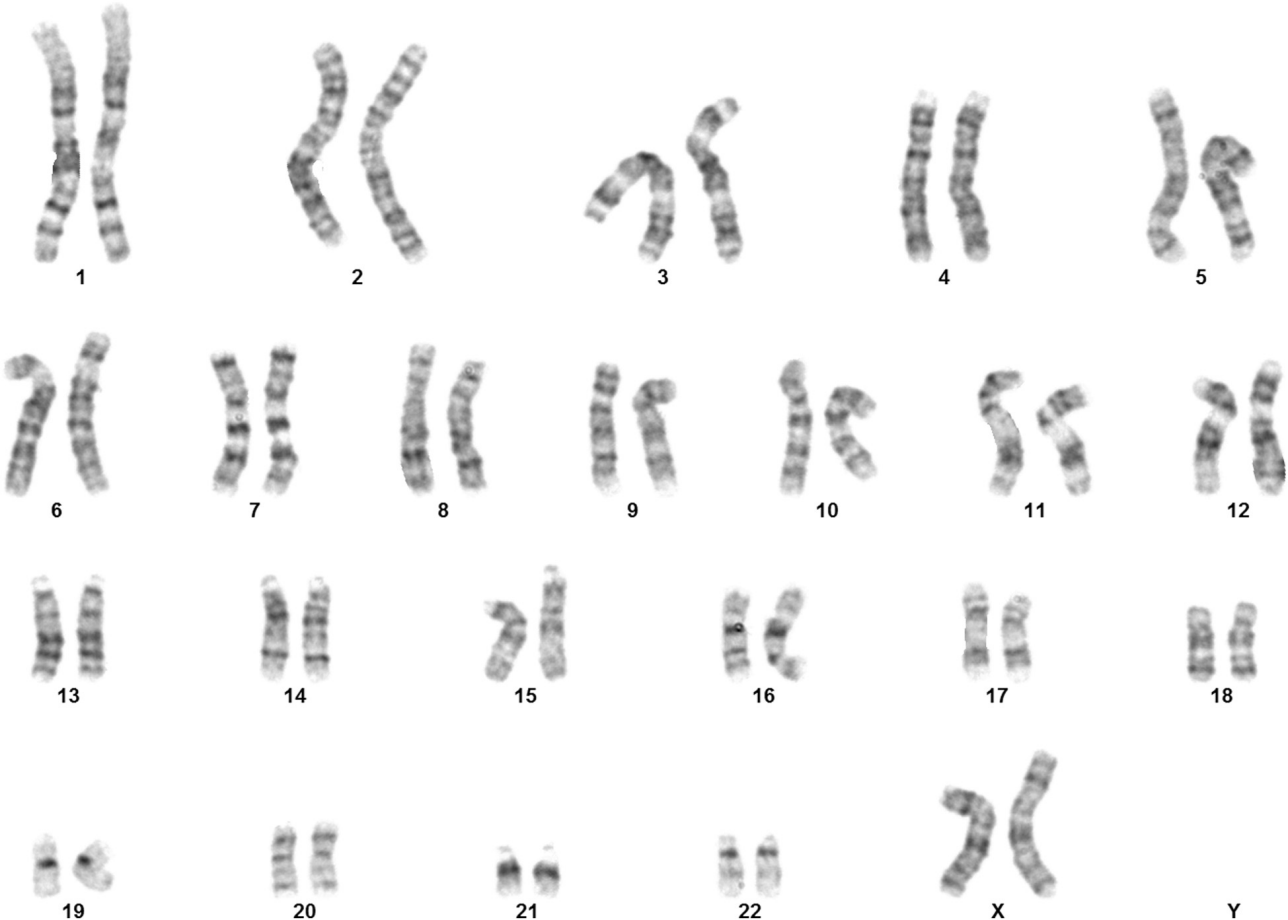
GTG banded Karyotype (Mother)

High resolution GTG banding performed on the couple and the affected daughter did not detect any chromosomal anomaly. The karyotype of the mother is shown in Fig. 2.

The FISH for chromosome 17 was performed by means of Miller-Dieker probe (Abbott) since this probe hybridizes with a region that contains the *PAFAH1B1* (*LIS1*) gene, located at 17p13.3, that is one of the causative genes for the lissencephaly phenotype in both Miller-Dieker syndrome (MDS) and Isolated lyssencephaly syndrome (ILS). The FISH for chromosome 5 was performed by means of subtelomeric probes for 5p (ToTelVysion from Vysis).

**Fig. 3:**
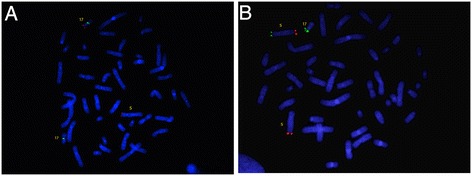
**a**: FISH for Miller Diecker Region on chromosome 17p: red probe LIS1 gene on 17p13.3, green probe RARA on 17q21.1(Mother). **b**: FISH with specific probes for chromosomes 5 p and q subtelomeric region: probe 5p15.33 (green), probe 5q35.3(red) (Mother)

FISH analysis of the father showed normal signals on both chromosomes 5 and 17 (data not shown), whereas FISH analysis of the mother showed two signals for the 17p, one located on the expected chromosome and the other on the short arm of chromosome 5 (5p) (Fig. 3a). When FISH was performed using the subtelomeric probes for 5p, two signals were detected: one on the expected chromosome and the other on the short arm of chromosome 17 (17p) (Fig. 3b). The analysis revealed a cryptic balanced translocation between chromosome 5 and chromosome 17. The karyotype of the mother was defined as 46,XX.ish t(5;17)(p15.3;p13.?2)(C84c11/T3-,+LIS1;-LIS1 + C84c11/T3).

**Fig. 4:**
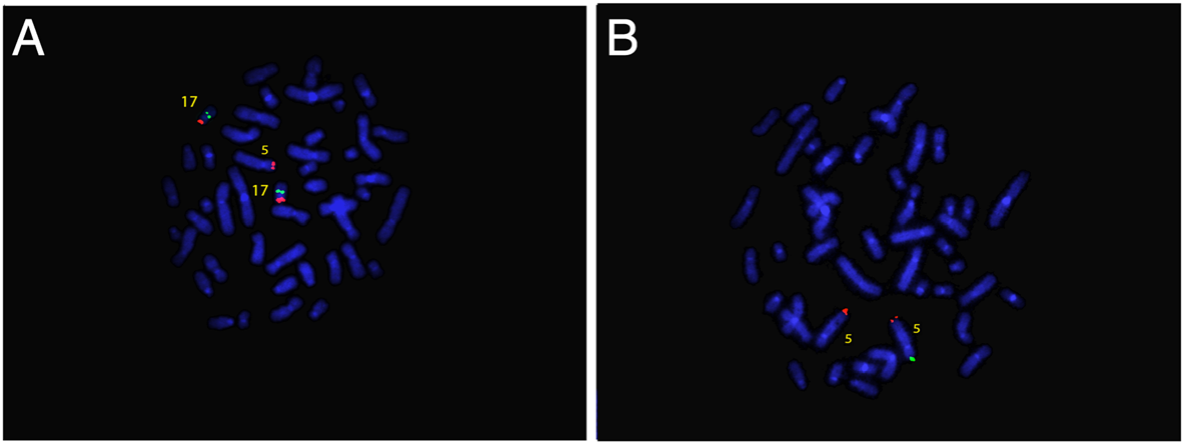
**a**: FISH for Miller Diecker Region on chromosome 17p: red probe LIS1 gene on 17p13.3, green probe RARA on 17q21.1 (Daughter). **b**: FISH with specific probes for chromosomes 5 p and q: green probe 5p15.33, red probe 5q35.3 (Daughter)

FISH analysis of the affected child revealed an unbalanced karyotype. When Abbott Miller-Dieker probe was used, three signals were detected: two on the 17p and one in 5p, revealing a partial trysomy for the region on 17p (Fig. 4a). When the 5p subtelomeric probe was used, only one signal for the 5p region was detected, confirming a partial monosomy on 5p (Fig. 4b). This result is consistent with maternal transmission of the unbalanced segregation adjacent-1.

**Fig. 5:**
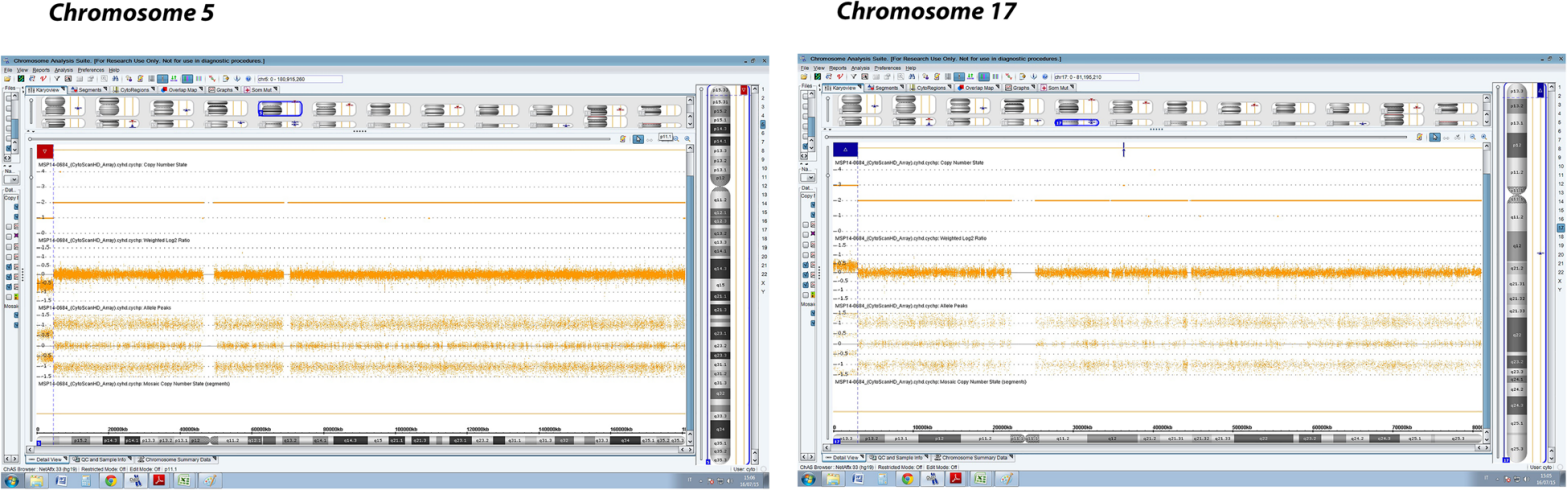
The results of SNP- array analysis analyzed and imaged using ChAS 2.0 software showing the deletion of the subterminal region of the short of a chromosome 5, of about 4.5 Mb, and the duplication of the subterminal region of the short arm of a chromosome 17, of about 3.1 Mb. Both log2 ratios and Allele Peaks calls indicated the locations and sizes of the chromosome anomalies

A SNP array performed on the DNA from the affected child (Fig. 5) confirmed the unbalanced karyotype and allowed an accurate determination of the breaking points. The karyotype of the affected child was defined as:

46,XX.ish der(5)t(5;17)(p15.32;p13.3)(C84c11/T3-,+LIS1;-LIS1 + C84c11/T3)mat.arr 5p15.33p15.32(113,576-4,612,673)×1,17p13.3(525-3,071,665)×3

The 5p (5p15.33p15.32) encompasses approximately 4,5 Mb and 21 genes described in OMIM database, 3 out of these genes are listed in the OMIM morbid map: *SDHA* (*600857), *SLC6A19* (608893) *TERT* (*187270).

The duplicated 17p13 region encompasses 38 genes described in OMIM database, 6 out of these genes are listed in the OMIM morbid map: *BHLHA*(*6I5416), *PRPF8* (*607300), *WDR81*(*614218), *SERPINF2*(*613168), *SERPINF1*(*172860), *PAFAH1B1*(*601545).

## Discussion

It is known that the terminal region of the short arm of chromosome 5 is involved in Cri du chat syndrome; nowadays the critical region has been located on chromosome 5p.15.2 [4].

More detailed studies have identified a 640 kb region included between the proximal part of 5p15.3 and the distal part of 5p15.2 as the ‘critical region’ for cat-like cry. A segment within 5p15.2 appears to be responsible for the ‘critical region’ for mental retardation [5, 6]. In addition, the distal portion of 5p15.3 has been related to speech delay [7, 8] although the association has not been confirmed in all the studies [9]. A recent study in CdCS patients suggests that haploinsufficiency of the telomerase reverse transcriptase (*hTERT*) gene, localized to 5p15.33, could contribute to the heterogeneous phenotype of CdCS. *hTERT* is the rate-limiting component for the telomerase activity that is essential for telomere-length maintenance and sustained cell proliferation [10, 11].

Table 1 compares dysmorphic features of our proband with phenotype attributable to sub terminal segmental deletion of 5p, as reported in the literature [7–9, 11–16], Cat like cry and, less consistently, delayed speech appear common features of these phenotypes.

**Table 1:** Comparison of Cri du chat clinical features among our case and others previously published (modified in part by [9])

Clinical features	Our case report	Elmakky et al. [9]					Van Buggenhout et al [8]	Rossi et al. [7]	Zhang et al. (2005)	Gersh et al. (1995)		Church et al. [11]	Cornish et al. (1999)	Kondoh et al. (2005)	Laczmanska et al (2006)
		III:1	III:2	III:3	III:4	III:5	Pt n°7	1 Patient	Pt n°117	Family 3 2 Patients	Family 4 4 Patients	5 Patients	4 Patients	Patient n°3	1 Patient
Chromosomal Anomaly	arr 5p15.33p15.32(113576-4612673)×1,17p13.3(525-3071665)×3	Der(5)t(5;15)(p15.3q11.1-2) with microdeletion involving 5p15.33-32					del(5)(p15.31)		del(5)(p15.3)						
Age	6y	7y	24 m	24 m	34y	58y	18y	35y	Nd	Nd	Nd	Nd	6-49y	6y	8d
Low birth weight	Nd	–	–	–	–	–	+	Nd	Nd	0/2	1/4	1/4	Nd	–	–
Growth retardation	Nd	+	+	+	+	–	–	–	Nd	0/2	1/4	1/4	Nd	+	Nd
Cat-like cry/high-pitched voice	+	+	+	+	+	+	+	+	+	2/2	3/4	3/4	0/5	–	+
Speech delay	+	–	–	–	–	–	–	+	+	0/2	0/4	0/4	5/5	Nd	Nd
ID	+	–	–	–	–	–	–	Mild	Mild	0/2	2/4 Mild	3/5 Mild	3/5 Mild	–	Nd
Motor delay	+	–	–	–	–	–	+	Nd	Nd	0/2	Nd	Nd	Nd	Nd	Nd
Neonatal hypotonia	–	–	+	+	–	Nd	+	Nd	Nd	0/2	1/4	Nd	Nd	–	–
Microcephaly	–	+	+	+	+	+	–	–	–	0/2	1/4	1/5	1/5	–	–
Dysmorphisms in childhood	+	Y	Y	Y	Nd	Nd	Y	Nd	N	Y/nd	N	N	N	N	Y
Small round face	–	+	+	+			+			–					+
Higly arched eyebrows	–	+	+	+			–			–					–
Synophris	+	+	+	+			–			–					–
Hypertelorism	+	–	–	–			+			–					–
Epicanthal folds	–	+	+	+			+			+					–
Strabismus	–	+	–	+			+			–					
Low set ears	+	–	–	–			–			+					+
Thin upper lip	+	+	+	+			–			–					–
Pointed chin	–	+	+	+			+			–					–
Single palmar creases	–	+	+	+			+			+					–
Disrmorphisms in adult	Nd	Nd	Nd	Nd	Y	Nd	Y	Y	N	Nd/N	N	N	N (1patient)	Nd	Nd
Others	clynodactily, hypothyroidism at birth	Flat feet	Hypospadia		Oligospermia	Flat feet	Metacarpals4-5th short, flat feet	Kydney Hypoplasia, azoospermia				Hypoacusis, preauricle skin tags			Cardiopathy, flat feet

17p13.3 microduplication syndrome is a rare clinical condition with a critical region overlapping the region deleted in Miller-Dieker lissencephaly syndrome (MDLS; 247200). A recently described duplication syndrome involving the 17p13.3 region has been associated with intellectual impairment, autism and occasional brain MRI abnormalities with large variability within and between families, particularly when cognitive development was considered [17, 18].

This variability certainly depends on how large the duplication is, how many genes it contains and what they do. Despite the variability of the clinical presentation, it is still possible to delineate a common clinical spectrum comprising mild to moderate psychomotor delay, hypotonia and discrete craniofacial dysmorphic features including a high forehead with frontal bossing, small nose and a small mouth [19].

Several genes map in 17p13.3 region [17, 18] 2 classes of microduplications of 17p13.3 have been described:

Class I duplications involve *YWHAE*, but not *PAFAH1B1*, whereas class II duplications involve *PAFAH1B1* and may also include *CRK* and *YWHAE*. Class I duplications are associated with autistic features and other behavioral symptoms, speech and motor delay, subtle dysmorphic facial features such as pointed chin and cupid bow, subtle hand/foot malformations, and a tendency to postnatal overgrowth. Class II microduplications are associated with moderate to mild developmental and psychomotor delay and hypotonia. Some of the dysmorphic features, such as prominent forehead and pointed chin, are shared with the class I duplications [17, 18].

The child presented in this case report shows features of the duplication syndrome involving the 17p13.3 region such as facial dysmorphisms (small mouth, subtle hand/foot malformations, speech and motor delay), as well as speech retardation that may also be attributed to 5p15.33 deletion.

Table 2 compares dysmorphic features of our proband with phenotype attributable to segmental duplication of 17p, as reported in the literature [17, 18, 20–23], Delayed speech as well as delayed mental development appear common features of these phenotypes.

**Table 2:** Comparison of 17p segmental duplication clinical features among our case and others previously published (modified in part by [23])

	Bi et al. Patient 7(2009)	Roos et al. Patient 1 (2009)	Roos et al. Patient 2 (2009)	Roos et al. Patient 3 (2009)	Bruno et al. Patient 10(2009)	Hyon et al. (2011)	Avela et al. (2011)	Ruiz Esparza-Garrido et al. (2012)	Our patient
Chromosomal abnormality	duplication	interstitial duplication	terminal duplication	terminal duplication	interstitial duplication	t(X;17)	ins(4;17)	t(10;17)	t(5;17)
Inheritance	de novo	de novo	de novo	de novo	de novo	?	de novo	from father	from mother
Size of duplication, Mb	3,6	1,8	3	4	2	4,2	7	3,22	3,1
Age at diagnosis, years	10	14	1	1	6,5	13	5	0,5	6
Sex	F	M	F	M	M	F	F	F	F
Birth Height, cm	53	53	NA	50	normal	normal	45	51	NA
Birth weigth, g	3060	3350	4200	3380	2670	normal	2570	3000	NA
Current Height	+1SD	+3,5SD	normal	+1SD	normal	+1SD	NA	50–75th percentile	111 cm (10–25th percentile)
Current weigth	+2SD	+1SD	−2SD	+1SD	−1,5SD	+1SD	NA	25th percentile	17 kg (10th percentile)
Craniofacial features									
Current head circumference	−1,5SD	NA	+	+2SD	−0,8 SD	+1SD	NA	37 cm (<3rd percentile)	50 cm (10–25th percentile)
Hypotonic face	–	+	+	+	NA	+	+	–	–
Broad midface	–	–	+	+	NA	+	+	–	+
Low seat ears	–	+	+	–	NA	–	+	–	+
Frontal bossing	–	+	+	+	NA	–	+	–	–
Triangular chin	NA	–	+	+	+	+	+	+	+
Downslanting palpebral fissures	–	+	+	–	+	+	+	–	+
Hypertelorism	–	+	+	+	–	+	+	+	+
Broad nasal bridge	–	+	+	+	–	+	+	+	+
Strabismus	+	–	–	–	–	–	–	+	–
Abnormalities of the philtrum and mouth appearance	normal	normal	small	small	prominent cupid bow	small	normal	normal	small
Neck appearance	normal	normal	short	short	normal	normal	short	+	normal
Cleft lip and palate	–	–	–	–	–	+	+	–	–
Clinodactyly	–	–	+	–	–	+	+	+	+
Hip luxation	–	–	–	+	–	–	NA	–	–
Equinovalgus	–	–	–	right	–	–	–	+	–
Hirsutism/Hypertrichosis	–	–	–	–	–	–	–	–	+
Neurological									
Hypotonia	–	+	+	+	+	+	+	–	–
Speech delay	+	+	+	+	–	+	+	+	+
Feeding difficulties	–	–	+	–	+	–	+	–	+
Delayed mental development	+	+	+	+	–	+	+	+	+
Abnormal behavior	+	+	+	+	autism	+	+	+	+
Hypothyroidism at birth	–	–	–	–	–	–	–	–	+

## Conclusion

Highly effective and sensitive genomic approaches are now available that overcome the limits of classic cytogenetic. Nevertheless the present case report suggests that a critical evaluation of both the pedigree and the reproductive history of the family is still extremely useful since it allows the formulation of clinical hypothesis that can be easily tested by FISH, the one and only technique that enables to see genes on chromosomes. Following this simple approach we were able to establish the recurrence risk of the couple and to re-evaluate the clinical phenotype of the child, by the extrapolation of the genomic data. Moreover a possible explanation was found for the lissencephaly described at least in one of the fetuses. Finally the appropriate diagnosis allowed the couple to consider a pre-implantation diagnosis based approach that would have been impossible before. We feel that subtelomeric FISH can still be suggested for couples with similar pedigree or with repeated miscarriage.

## Consent

Written informed consent was obtained from patient's parents for the publication of this report and any accompanying images.
